# The effect of different deproteinization agents and thermal cycling on the shear bond strength of fissure sealants: an in vitro study

**DOI:** 10.1186/s12903-026-08167-z

**Published:** 2026-03-27

**Authors:** Merve Tatar, Alem Coşgun

**Affiliations:** Department of Pediatric Dentistry, Faculty of Dentistry, Tokat Gaziosmanpaşa University, Tokat, 60100 Türkiye

**Keywords:** Enamel, Shear Bond Strength, Hypochlorous acid, Sodium hypochlorite, Bromelain

## Abstract

**Background:**

Enamel pretreatment with a deproteinization agent enhances fissure sealant bonding. This in vitro study investigates the effect of surface preparation methods and thermal cycling on the shear bond strength of fissure sealants applied to permanent teeth.

**Methods:**

Sixty sound human third molars were randomly divided into ten groups (*n* = 6). Groups 1–5 received different surface treatments (control, HOCl 15 s, HOCl 30 s, NaOCl 60 s, and bromelain (plant-based agent) 60 s), while Groups 6–10 were subjected to identical protocols with the addition of 10,000 thermal cycles. All samples underwent shear bond strength testing. Statistical analysis was performed using IBM SPSS Statistics version 23.0 (IBM Corp., Armonk, NY, USA), with *p* < 0.05 considered statistically significant.

**Results:**

Surface preparation methods had no significant effect on shear bond strength (*p* = 0.431), while thermal cycling significantly reduced bond strength across all groups (*p* = 0.022). No interaction was found between the two factors (*p* = 0.480). A decreasing trend was observed within thermocycled groups: sodium hypochlorite > bromelain > hypochlorous acid (30 s) > hypochlorous acid (15 s) > control.

**Conclusions:**

Within the limitations of this in vitro study, surface deproteinization did not statistically significantly increase shear bond strength compared to the control group. However, deproteinization agents showed a defining trend toward better preservation of bond strength stability following thermal cycling. Given their biocompatibility and favorable performance, bromelain and hypochlorous acid can be considered promising alternatives to sodium hypochlorite, but further clinical studies are needed to optimize application protocols and validate their long-term efficacy in pediatric dentistry.

## Background

Various preventive dentistry approaches are employed to inhibit the development of dental caries. Among these, pit and fissure sealants have long been considered an effective method for preventing caries formation on at-risk tooth surfaces and for arresting the progression of early enamel lesions [1, 2]. However, the long-term clinical success of these sealants depends heavily on the quality and durability of the bond between the material and the enamel surface. Acid etching of the enamel surface is a conventional, standard, and essential step in the pretreatment process of pit and fissure sealant applications [3]. This procedure creates microporosities on the enamel surface to enhance the retention of the sealant, increase enamel wettability, and provide an antibacterial effect. However, organic debris within the fissures may limit acid penetration and effectiveness, thereby hindering the formation of an optimal surface for adhesion [4, 5]. To overcome this biological barrier, removing organic matter from the enamel surface prior to acid etching has been proposed to enable more effective surface roughening. This step not only enhances bond strength but also optimizes the adhesion and retention of adhesive materials to tooth structure, thereby achieving more durable and effective retention. To improve the clinical success of fissure sealants, several surface preparation methods have been proposed, including pumice prophylaxis, air abrasion, air polishing, laser treatment, and deproteinization. It has been demonstrated that applying a deproteinization agent to the enamel surface as a pretreatment step increases the bond strength of fissure sealants to enamel [6, 7].

Traditionally, sodium hypochlorite (NaOCl) at a concentration of 5.25% has been reported in numerous studies to be effective as an enamel deproteinization agent, removing organic components of the pellicle from the enamel structure and surface prior to acid application, thereby improving adhesion. However, due to its strong oxidizing properties, sodium hypochlorite poses potential risks for soft tissue reactions, particularly in pediatric dentistry applications. Its unpleasant taste and odor have prompted the search for alternative materials that can provide similar proteolytic benefits with improved biocompatibility [6–9].

One such alternative is hypochlorous acid (HOCl) is a highly effective oxidizing agent against bacteria, viruses, and fungi. Widely utilized in fields such as food safety, water purification, medical applications, and agriculture, this compound also plays a significant role in dentistry due to its antimicrobial activity and deproteinization properties [10–12].

In addition to synthetic agents, natural alternatives have also been explored; for instance, bromelain is a natural enzyme complex derived from the fruit or stem of the pineapple plant. As a plant-based agent, it exhibits various therapeutic effects, including inhibition of platelet aggregation, fibrinolytic activity, anti-inflammatory action, and both bacteriostatic and bactericidal properties. Although bromelain has long been used in the medical and food industries, it is relatively new to the field of dentistry. Due to its anti-inflammatory and deproteinization properties, this enzyme has gained increasing attention in dental applications [13, 14].

Several promising studies have explored hypochlorous acid and bromelain as potential alternatives to sodium hypochlorite for the deproteinization of enamel [15–18]. While these initial bond strength outcomes are promising, the intraoral longevity of these treatments under functional stresses remains a critical concern. Thermal cycling is an artificial aging method used to simulate the thermal and mechanical stresses to which restorations are subjected in the oral environment over time. Under in vitro conditions, alternating temperature and load are applied to the restorations to replicate the challenges encountered intraorally. Based on the ISO TR 11,450 guidelines, an artificial aging test involving 500 thermal cycles between 5 °C and 55 °C is regarded as an optimal protocol for simulating clinical conditions [19–21]. Gale and Darvell reported that the application of 10,000 thermal cycles simulates approximately one year of intraoral function under clinical conditions [19].

By combining these various surface treatments with an aging protocol, a more comprehensive understanding of their clinical potential can be achieved. While deproteinization has been previously explored, the search for agents that balance proteolytic efficacy with clinical safety remains a priority. This study uniquely evaluates HOCl and Bromelain under standardized aging conditions, providing essential data for their potential integration into daily pediatric dental practice. Therefore, the aim of this study is to evaluate, in vitro, the effect of different surface preparation methods combined with thermal cycling on the shear bond strength of fissure sealants applied to permanent teeth. The first null hypothesis (H₀₁) of the study states that ‘Different surface preparation methods do not have a statistically significant effect on the shear bond strength of fissure sealants‘. The second null hypothesis (H₀₂) states that ‘Thermal cycling does not have a statistically significant effect on the shear bond strength of fissure sealants‘.

## Materials and methods

### Ethical approval

This study was approved by the Non-Interventional Scientific Research Ethics Committee of the Faculty of Medicine at Tokat Gaziosmanpaşa University (Approval Date: January 21, 2025; Reference No: 25-MOBAEK-031). The study was conducted in accordance with the ethical principles of the Declaration of Helsinki. This laboratory-based study was conducted in compliance with the RoBDEMAT guidelines.

### Power analysis

To estimate the required sample size, G*Power version 3.1.9.4 software was used with the F-test family in a fixed-effects ANOVA model. For 10 groups, with an effect size of 0.82, a statistical power of 80%, and an alpha level of 0.05, the minimum required sample size was calculated as 40 (i.e., at least 4 specimens per group) [22]. Considering the possibility of sample loss, the study was conducted on a total of 60 specimens, with 6 assigned to each group.

### Study design and tooth selection criteria

This study was conducted under in vitro conditions. A total of 60 extracted human mandibular third molars with an ICDAS score of 0, obtained during routine clinical extractions at the Department of Oral and Maxillofacial Surgery, XXX University, following informed consent from all patients, were included in the study. Exclusion criteria comprised teeth with fractures or cracks, carious lesions, restorations, malformations, enamel hypomineralization, extraction-related defects on the enamel surface, or any signs of erosion.

### Collection and preparation of teeth used in the study

The teeth were stored in distilled water at + 4 °C, which was refreshed weekly, and were used within a maximum of three months. Prior to the experiment, all teeth were thoroughly cleaned to remove any residues and embedded in cold-cure acrylic resin with the buccal surfaces exposed. To obtain a flat and standardized surface, the exposed enamel was ground for 10 s under water cooling using 600-grit silicon carbide abrasive paper in a horizontal rotary polishing machine (Bulupol-1 Metallographic Specimen Grinding & Polishing Machine, BMS Bulut Makina Sanayi Ve Ticaret Ltd. Şti., Türkiye).

Subsequently, six healthy volunteers (2 males, 4 females), aged between 22 and 32 years, who were physicians or dental interns at our clinic, and who had no systemic diseases, were not using orthodontic appliances, did not smoke, had no hyposalivation, and were not pregnant or lactating, were verbally informed and signed an informed consent form. From each participant, at least 12 milliliters of unstimulated saliva were collected into sterile containers. To minimize biological variability and ensure a standardized application across all experimental groups, the collected saliva samples were pooled into a single sterile container and gently homogenized [23]. The pH of the pooled saliva was measured using a digital pH meter (pH range: 6.9–7.2) to ensure it remained within the physiological range. The freshly pooled saliva was applied to the tooth surfaces using an applicator for 1 min, followed by rinsing and drying for 10 s. This procedure was performed to simulate the oral environment. 

**Fig. 1:**
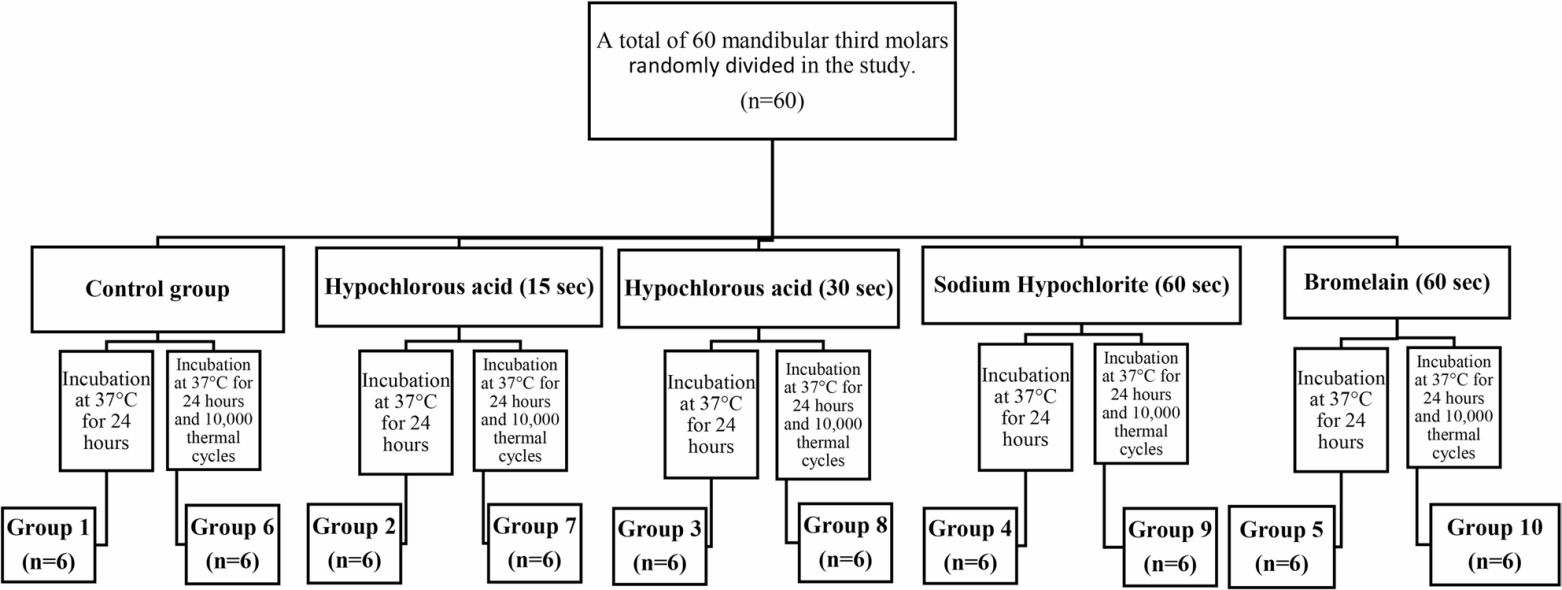
Study Groups

**Fig. 2:**
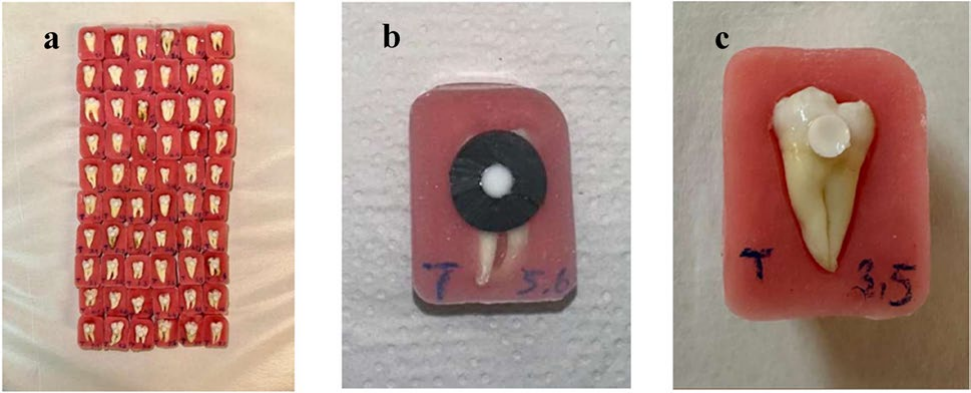
Preparation of Experimental Specimens

The experimental groups formed for the study are presented in Fig. 1. The teeth were randomly assigned to the groups. All restorations were performed by a single trained operator (M.T.). The preparation of the specimens used in the study is illustrated in Fig. 2. The materials used in the study, along with their properties, compositions, and manufacturers, are listed in Table 1.

**Table 1:** Materials used in the study

Material	Property	Composition	Manufacturer
3 M Clinpro™ Sealant	Fissure sealant material	Bis-GMA, TEGDMA, silane, tetrabutylammonium tetrafluoroborate, diphenyl hexafluorophosphate, EDMAB, titanium hydroxide, hydroquinone	3 M, St. Paul, MN, USA
K-ETCHANT Syringe Etchant	Etching	37% phosphoric acid	K-ETCHANT, Syringe, Kuraray Kuraray Noritake Dental Inc., Tokyo, Japan
Sodium hypochlorite solution	Deproteinizing agent	5.25% NaOCl	Wizard Sodium Hypochlorite, Atlas Dental Supply, Türkiye
Hypochlorous acid	Deproteinizing agent	pH 6.2, 200 ppm available chlorine	Superox, Anolit Hygiene and Chemical Industry Inc., Ankara, Türkiye
Bromelain powder extract	Plant-based deproteinizing agent	50 g bromelain powder extract	Nurbal Şifa Aktar Natural Food Industry Ltd., Istanbul, Türkiye

In this study, bromelain was used as a deproteinization agent in its commercially available powder form, as no ready-to-use solution or gel formulation is currently produced. Based on similar reports in the literature, a 10% bromelain solution was prepared by dissolving 10 g of bromelain powder in 100 milliliters of distilled water [18, 24].

- *Group 1 (Control),* 37% phosphoric acid (K-ETCHANT, Syringe, Kuraray Noritake Dental Inc., Tokyo, Japan) was applied to the buccal enamel surfaces for 30 s, followed by rinsing and drying. A plastic cylindrical mold with an inner diameter of 4 mm and a height of 2 mm was then placed on the prepared surface. To prevent leakage, the outer edges of the mold were sealed with a gingival barrier. The fissure sealant material, Clinpro™ Sealant (3 M, St. Paul, MN, USA), was applied inside the mold to a height of 2 mm and polymerized for 20 s using a LED light-curing device (Elipar S10, 3 M ESPE).
- *Group 2 (HOCl-15 s),* 200 ppm hypochlorous acid (Superox, Anolit Hygiene and Chemical Industry Inc., Ankara, Türkiye) was applied to the buccal surfaces of the teeth for 15 s, followed by rinsing and drying. Subsequently, phosphoric acid etching and fissure sealant application were performed as in the control group.
- *Group 3 (HOCl-30 s),* 200 ppm hypochlorous acid (Superox, Anolit Hygiene and Chemical Industry Inc., Ankara, Türkiye) was applied to the buccal surfaces of the teeth for 30 s, followed by rinsing and drying. Subsequently, phosphoric acid etching and fissure sealant application were performed as in the control group.
- *Group 4 (NaOCl),* 5.25% sodium hypochlorite (Wizard Sodium Hypochlorite, Atlas Dental Supply, Türkiye) was applied to the buccal surfaces of the teeth for 60 s, followed by rinsing and drying. Subsequently, phosphoric acid etching and fissure sealant application were performed as in the control group.
- *Group 5 (Bromelain),* 10% bromelain solution was applied to the buccal surfaces of the teeth for 60 s, followed by rinsing and drying. Subsequently, phosphoric acid etching and fissure sealant application were performed as in the control group.
- *Group 6,* the same procedural steps as Group 1, were applied to the buccal surfaces of the teeth. Following this, the specimens underwent artificial aging through 10,000 thermal cycles between 5 °C and 55 °C to simulate oral conditions, using a thermal cycling device (Thermal Cycling Machine, Moddental, Ankara, Türkiye).
- *Group 7,* the same procedural steps as Group 2, were applied to the buccal surfaces of the teeth. Following this, the specimens underwent artificial aging through 10,000 thermal cycles between 5 °C and 55 °C, performed with a thermal cycling device (Thermal Cycling Machine, Moddental, Ankara, Türkiye), in order to simulate the oral environment.
- *Group 8,* the same procedural steps as Group 3, were applied to the buccal surfaces of the teeth. Following this, the specimens underwent artificial aging through 10,000 thermal cycles between 5 °C and 55 °C using a thermal cycling machine (Moddental, Ankara, Türkiye) to simulate the oral environment.
- *Group 9,* the same procedural steps as Group 4, were applied to the buccal surfaces of the teeth. Subsequently, the specimens underwent artificial aging through 10,000 thermal cycles between 5 °C and 55 °C to simulate the oral environment, using a thermal cycling machine (Moddental, Ankara, Türkiye).
- *Group 10,* the same procedural steps as Group 5, were applied to the buccal surfaces of the teeth. Subsequently, to simulate the oral environment, the specimens underwent artificial aging through 10,000 thermal cycles between 5 °C and 55 °C using a thermal cycling machine (Moddental, Ankara, Türkiye).

### Measurement of shear bond strength

All specimens were incubated in a humid environment at 37 °C for 24 h (Nüve Incubator, EN 055, Ankara, Türkiye). Subsequently, to simulate oral conditions, the samples in Groups 6, 7, 8, 9, and 10 underwent artificial aging through 10,000 thermal cycles between 5 °C and 55 °C (dwell time: 15 s, transfer time between baths: 5 s) using a thermal cycling machine (Moddental, Ankara, Türkiye). Shear bond strength testing was performed on all specimens using the standardized Ultradent method with a universal testing machine (Lloyd 1 KN, Ametek Ltd., USA), applying force at a crosshead speed of 1 mm/min. The debonding force for each specimen was recorded in Newtons (N), and the bond strength was calculated in megapascals (MPa) by dividing the force by the bonding area (N/mm²).

### Stereomicroscopic examination of the specimens

Following the test, the enamel surfaces were examined at 40× magnification using a stereomicroscope (Stereo Microscope, Olympus SZ61, Japan) to assess the failure mode. Micrographs of the specimens were captured using a camera system attached to the microscope, and the failure types were classified by a single trained operator (M.T.) based on these images. The failure modes were categorized as follows:

1. Enamel fracture: Failure occurs within the enamel surface.
2. Adhesive failure: Failure occurs at the interface between the enamel surface and the fissure sealant.
3. Cohesive failure: Failure occurs within the fissure sealant material.
4. Mixed failure: Failure involves both the adhesive interface and the sealant material.

### Statistical analysis

The data were analyzed using IBM SPSS Statistics Version 23.0 (IBM Corp., Armonk, NY, USA). The normality of the data distribution was assessed using the Kolmogorov–Smirnov test. For normally distributed shear bond strength values, two-way ANOVA was employed to compare the effects of different surface preparation methods and thermal cycling application. Descriptive statistics for quantitative variables were presented as mean ± standard deviation. The distribution of failure modes among the groups was compared using Fisher’s Exact Test with Monte Carlo correction. Descriptive statistics for categorical variables were expressed as frequencies and percentages. Pairwise comparisons of proportions were conducted using the Bonferroni-corrected Z test. A significance level of *p* < 0.05 was considered statistically significant.

## Results

The main effect of different surface preparation methods on the mean shear bond strength was not statistically significant (*p* = 0.431). However, the main effect of thermal cycling on the mean shear bond strength was statistically significant (*p* = 0.022). The interaction between surface preparation methods and thermal cycling did not result in a statistically significant difference in the mean shear bond strength values (*p* = 0.480). A detailed comparison of the effects of surface preparation methods and thermal cycling on shear bond strength is presented in Table 2.

**Table 2:** Effects of surface preparation methods and thermal cycling on shear bond strength

	Sum of Squares	Degrees of Freedom	Mean Square	F	*p*	η^2^
Surface Preparation Method	62.016	4	15.504	0.973	0.431	0.072
Thermal Cycling	88.552	1	88.552	5.557	0.022	0.100
Surface Preparation Method*Thermal Cycling	56.382	4	14.096	0.885	0.480	0.066
Error	796.739	50	15.935			
General	5333.199	60				

F: Analysis of Variance (ANOVA) Test Statistic; η^2^: partial eta squared; R^2^: 20.6%; Adjusted R^2^: 6.3%

**Fig. 3:**
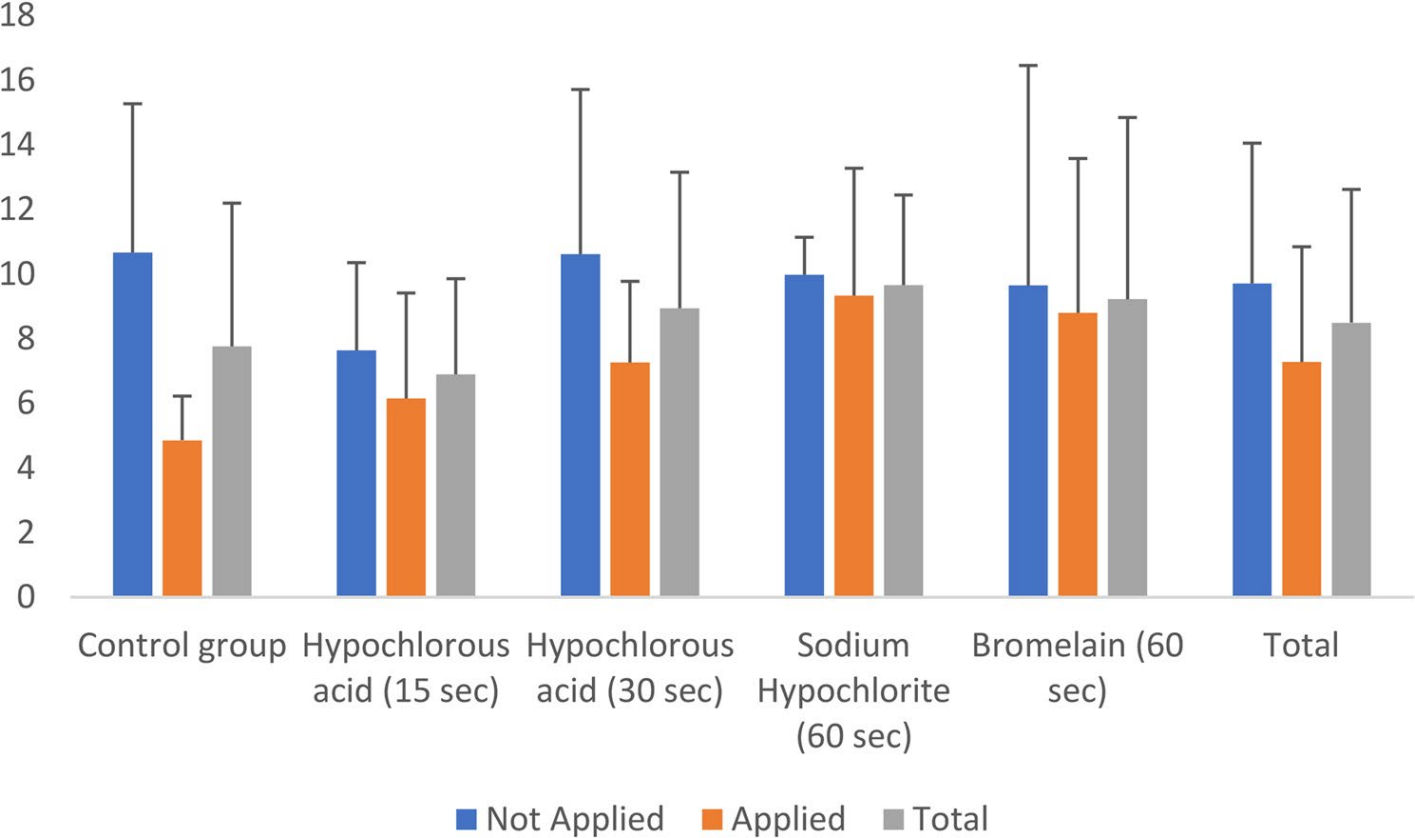
Shear Bond Strength Across Surface Preparation Methods and Thermal Cycling

In the control group, which did not receive any deproteinization agent, the mean shear bond strength was 7.76 ± 4.44. The group treated with 200 ppm hypochlorous acid for 15 s showed a mean shear bond strength of 6.90 ± 2.96, while the group treated for 30 s had a mean value of 8.94 ± 4.21. The group treated with 5.25% sodium hypochlorite (60 s) exhibited a mean shear bond strength of 9.66 ± 2.79, and the group treated with 10% bromelain (60 s) showed a mean value of 9.22 ± 5.62. No statistically significant difference was found among the ten groups resulting from the interaction effect. Descriptive statistics for shear bond strength values across different surface preparation methods and thermal cycling conditions are presented in Table 3 Fig. 3

**Table 3:** Descriptive statistics of shear bond strength for different surface preparation methods and thermal cycling

Surface Preparation	Thermal Cycling			Total
	Not Applied		Applied	
Control group	10.67 ± 4.6	4.86 ± 1.36		7.76 ± 4.44
Hypochlorous acid (15 s)	7.64 ± 2.71	6.15 ± 3.26		6.9 ± 2.96
Hypochlorous acid (30 s)	10.61 ± 5.09	7.26 ± 2.51		8.94 ± 4.21
Sodium Hypochlorite (60 s)	9.98 ± 1.16	9.33 ± 3.94		9.66 ± 2.79
Bromelain (60 s)	9.64 ± 6.81	8.8 ± 4.78		9.22 ± 5.62
Total	9.71 ± 4.34	7.28 ± 3.56		8.49 ± 4.12

Mean ± Standard Deviation (SD)

Among the groups without thermal cycling, the mean shear bond strength values in descending order were as follows: control group > hypochlorous acid (30 s) > sodium hypochlorite (60 s) > bromelain (60 s) > hypochlorous acid (15 s). In contrast, in the thermally cycled groups, the descending order was: sodium hypochlorite (60 s) > bromelain (60 s) > hypochlorous acid (30 s) > hypochlorous acid (15 s) > control group.

No statistically significant difference was observed in the distribution of failure modes among the groups without thermal cycling (*p* = 0.565). In Group 1, the proportions of failure types were as follows: enamel fracture 33.3%, adhesive failure 0%, cohesive failure 16.7%, and mixed failure 50%. In Group 2: enamel fracture 33.3%, adhesive failure 16.7%, cohesive failure 16.7%, and mixed failure 33.3%. In Group 3: enamel fracture 33.3%, adhesive failure 16.7%, cohesive failure 16.7%, and mixed failure 33.3%. In Group 4: enamel fracture 0%, adhesive failure 33.3%, cohesive failure 0%, and mixed failure 66.7%. In Group 5: enamel fracture 0%, adhesive failure 50%, cohesive failure 0%, and mixed failure 50%.

**Fig. 4:**
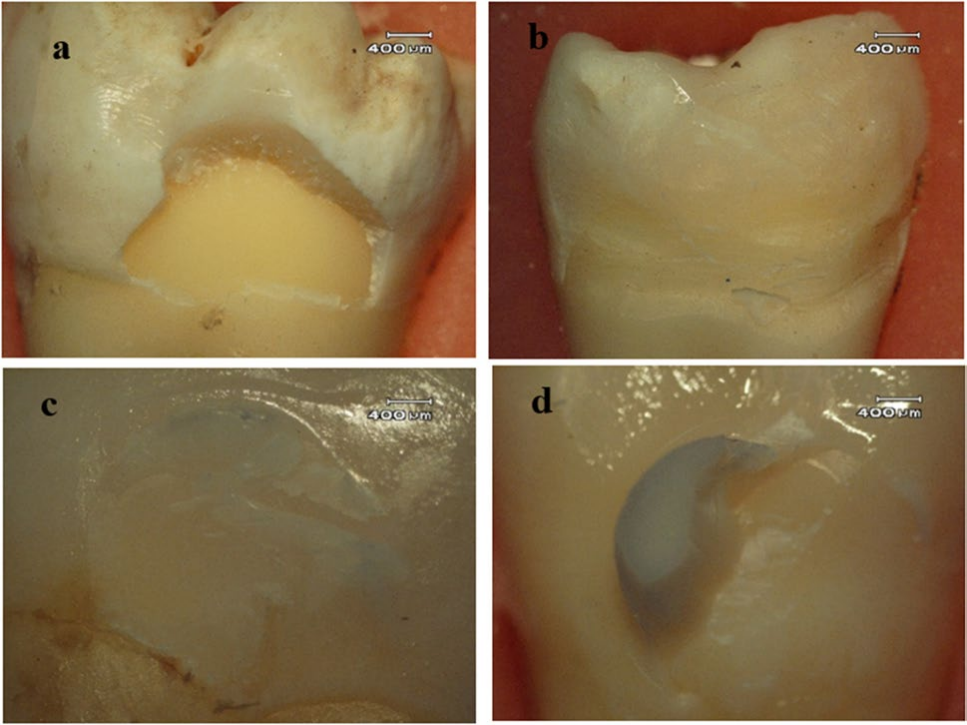
Microscopic images of fracture types. **a** Specimen with enamel fracture, **b** Specimen with adhesive failure, **c** Specimen with cohesive failure, **d** Specimen with mixed failure

In contrast, a statistically significant difference was found in the distribution of failure modes among the groups that underwent thermal cycling (*p* = 0.003). In Group 6, enamel fracture occurred in 50% of cases, adhesive failure in 33.3%, and mixed failure in 16.7%. In Group 7, adhesive failure accounted for 100%, with no enamel fracture or mixed failure observed. In Group 8, enamel fractures in 66.7% and mixed failures in 33.3%. In Group 9: enamel fracture 66.7%, adhesive failure 16.7%, and mixed failure 16.7%. In Group 10: enamel fracture 16.7%, adhesive failure 16.7%, and mixed failure 66.7%. A significant difference in adhesive failure distribution was observed between Group 7 and Groups 8, 9, and 10. The comparison of failure modes according to thermal cycling conditions is presented in Table 4. Representative stereomicroscopic images of the failure types are shown in Fig. 4.

**Table 4:** Fracture type comparison across thermal cycling conditions

Thermal Cycling	Group	Enamel fracture	Adhesive failure	Cohesive failure	Mixed failure	Test Statistic	*p*
Not Applied	Group 1	2 (33.3)	0 (0)	1 (16.7)	3 (50)	10.564	0.565^x^
	Group 2	2 (33.3)	1 (16.7)	1 (16.7)	2 (33.3)		
	Group 3	2 (33.3)	1 (16.7)	1 (16.7)	2 (33.3)		
	Group 4	0 (0)	2 (33.3)	0 (0)	4 (66.7)		
	Group 5	0 (0)	3 (50)	0 (0)	3 (50)		
Applied	Group 6	3 (50)	2 (33.3)^ab^	-	1 (16.7)	18.243	**0.003** ^**x**^
	Group 7	0 (0)	6 (100)^b^	-	0 (0)		
	Group 8	4 (66.7)	0 (0)^a^	-	2 (33.3)		
	Group 9	4 (66.7)	1 (16.7)^a^	-	1 (16.7)		
	Group 10	1 (16.7)	1 (16.7)^a^	-	4 (66.7)		

^x^ Monte Carlo Corrected Fisher’s Exact Test; n(%)

^a−b^ Means with the same letter are not significantly different

## Discussion

The long-term success of fissure sealants depends heavily on achieving a durable, stable bond to the enamel surface, which serves as a critical barrier against caries. However, the presence of organic pellicles and debris within pits and fissures can reduce acid etching efficiency, potentially compromising marginal integrity and sealant retention over time. Enamel deproteinization has emerged as a promising pretreatment to overcome these challenges by removing organic contaminants before bonding. The primary objective of this study was to evaluate the comparative efficacy of conventional (NaOCl) and alternative, biocompatible deproteinization agents (HOCl and Bromelain) under thermal cycling stress to simulate the dynamic intraoral environment. By understanding how these agents influence shear bond strength, we aimed to identify viable alternatives that maintain clinical performance while improving patient safety and comfort. In light of these objectives, the first null hypothesis -‘Different surface preparation methods do not have a significant effect on the shear bond strength of fissure sealants’- was supported by the findings. However, the second null hypothesis -‘Thermal cycling does not have a significant effect on the shear bond strength of fissure sealants’- was rejected.

To the best of our knowledge, no previous study in the literature has simultaneously compared the effects of NaOCl, HOCl, and bromelain solutions as deproteinization agents on enamel surfaces. Therefore, this study is the first to address this comparison within a single experimental design.

The outcomes of this study underscore the detrimental impact of artificial aging on the adhesive interface’s stability. Regardless of the surface treatment applied, thermal cycling significantly compromised the shear bond strength of the fissure sealants, a finding that reflects the inevitable hydrolytic degradation and stress-induced fatigue that occur in the oral environment. Notably, the control group, which achieved the highest initial bond strength, experienced the most substantial decline following thermal aging, ultimately resulting in the lowest values among all groups. This pattern suggests that while a standard acid-etching protocol may provide sufficient immediate adhesion, it may lack the necessary resilience to withstand long-term dynamic stresses. In contrast, the numerical preservation of bond strength observed in the deproteinization groups, though not reaching statistical significance, indicates that removing organic content from the enamel may create a more durable and stable morphologic substrate. While these descriptive trends must be interpreted with caution due to the lack of statistical superiority among the agents, they suggest that deproteinization before sealant placement could be a protective measure, enhancing the longevity and stability of the bond under simulated clinical conditions [6, 9, 25, 26].

A meta-analysis testing the hypothesis of whether deproteinization agents should be applied before or after acid etching found that when these agents are used prior to acid etching, they enhance the bond strength of various resin-based materials to enamel surfaces. In contrast, no significant effect was observed when applied after acid etching [27]. Therefore, in our study, deproteinization agents were applied to the enamel prior to acid etching. However, in contrast to our results, a study in the literature reported that deproteinization agents had no enhancing effect on the shear bond strength of composite resins. We believe that this discrepancy may be attributed to the application of the deproteinization agents after acid etching in that particular study [28].

Moreover, studies reporting that the application of deproteinization agents does not improve bond strength, and instead consider it a time-consuming additional step, were found not to include thermal cycling in their methodologies [8, 29]. In evaluating the results of our study, consistent with previous findings, the highest shear bond strength was observed among the groups without thermal cycling in the control group, which did not receive any deproteinization agent. However, following thermal cycling, all groups treated with deproteinization agents exhibited numerically higher, though not statistically, different bond strength values compared to the thermally cycled control group. Therefore, we believe that this difference may be attributed to the absence of thermal cycling in certain studies, although our statistical model did not identify surface treatment as a significant independent factor.

In our study, the group treated with NaOCl exhibited the least reduction in shear bond strength following thermal cycling, suggesting that NaOCl may be the most effective deproteinization agent. Although the shear bond strength values for the thermally cycled bromelain group were not statistically significant, they were still higher than those of the thermally cycled control group. In contrast to our findings, studies by Khatib et al. and Chauhan et al. reported higher bond strength values for bromelain compared to NaOCl. However, it should be noted that both of these studies evaluated deproteinization on dentin surfaces, not enamel [30, 31]. We believe that this discrepancy may be attributed to structural differences between enamel and dentin. However, in a study by Hasija et al., which investigated the effect of various deproteinization agents on the bond strength of composite resin to primary tooth enamel, it was reported that, although not statistically significant, bromelain gel resulted in higher bond strength compared to the control group, NaOCl, and papain [15]. Primary teeth have a higher organic content compared to permanent teeth; therefore, the discrepancies observed in the literature may be attributed to the fact that studies are often conducted on primary teeth. When evaluating these results, it is essential to note that NaOCl is a potent chemical agent with undesirable side effects, including an unpleasant taste, odor, and adverse tissue reactions. In contrast, bromelain has shown promising outcomes as a biocompatible alternative.

Another deproteinization agent considered as an alternative to NaOCl is HOCl. Although the difference was not statistically significant, the HOCl-30 group demonstrated higher bond strength values compared to the control groupIn the only study found in the literature in which hypochlorous acid was applied as a deproteinization agent on enamel, findings similarly revealed that both NaOCl (60 s) and HOCl (30 s) yielded statistically similar bond strength values to each other, a finding that is consistent with the outcomes of our current study [17].

In our study, failure types were classified into four categories: enamel fracture, adhesive failure, cohesive failure, and mixed failure [22, 32]. In the present study, the significant divergence in failure patterns following thermal aging-particularly the exclusive occurrence of adhesive failures in the HOCl-15 group-warrants detailed interpretation. This shift suggests that a 15-second application of hypochlorous acid may be insufficient to thoroughly deproteinize the enamel surface, potentially leaving residual organic remnants that impede the formation of high-quality resin tags. When the organic pellicle is not fully removed, the subsequently applied acid etchant cannot create the ideal microporosity required for a strong mechanical interlock. Consequently, the bond becomes highly susceptible to hydrolytic degradation at the interface during thermal cycling, leading to the observed adhesive failures where the sealant separates cleanly from the tooth. In contrast, the presence of mixed and cohesive failures in the HOCl-30, NaOCl, and Bromelain groups indicates a more integrated bond capable of transferring stress through the material or the tooth structure itself. This suggests that while HOCl is a potent agent, its efficacy in enhancing bond durability is time-dependent, with longer application times potentially facilitating a more favorable substrate for long-term clinical retention.

Organic residues present in fissures may limit acid penetration and effectiveness, thereby preventing the formation of optimal surface conditions required for adhesive bonding. For this reason, various studies have evaluated the efficacy of deproteinization agents. However, a review of the literature reveals that most existing in vitro studies have been conducted on teeth with fully cleaned enamel surfaces. Evaluating the effectiveness of deproteinization agents on such completely cleaned surfaces may not accurately reflect true clinical conditions and may thus be considered methodologically inappropriate. Therefore, in our study, the tooth surfaces were initially cleaned to ensure standardization during specimen preparation. To better simulate the oral environment, saliva was subsequently applied to the tooth surfaces, followed by rinsing and drying. This approach, designed to assess the effectiveness of deproteinization agents, is considered novel as it more realistically mimics intraoral conditions.

The primary limitation of this study is that it was conducted under in vitro conditions. Although in vitro studies offer certain advantages, and thermal cycling can mimic intraoral conditions to some extent, there remains a need for clinical trials. Furthermore, the use of flattened enamel surfaces to standardize the bonding area represents a significant limitation. While this approach allows for a controlled evaluation of shear bond strength, it does not fully reflect the natural and complex morphology of pits and fissures where sealants are clinically applied. Additionally, this study only included extracted teeth without enamel hypomineralization, which represents another limitation. Further research is needed to evaluate the effects of enamel deproteinization in teeth with altered enamel structures, such as those affected by amelogenesis imperfecta or molar incisor hypomineralization (MIH). The findings of this study should be interpreted with caution, taking into account the modest adjusted R^2^ value (6.3%). This statistical outcome indicates that the variables studied (surface preparation method and thermal cycling) account for only a small portion of the total variance in shear bond strength, suggesting that other unmeasured clinical and material-related factors also play a significant role in the bonding process. Therefore, future long-term clinical studies are recommended to evaluate additional variables that may affect the bonding performance of fissure sealants.

## Conclusions

Within the limitations of this in vitro study, although surface preparation methods did not show a statistically significant difference overall, deproteinization agents showed a descriptive trend towards preserving bond strength better than the control group after thermal aging. Given their biocompatible nature and the favorable shear bond strengths observed in this study, bromelain and hypochlorous acid could be considered potential alternatives to sodium hypochlorite. Although bromelain is biocompatible, the lack of readily available commercial solutions or gel formulations underscores the need for further research exploring different concentrations and application times. While hypochlorous acid shows promise due to its availability as a prepared solution, long-term clinical studies are required to confirm its efficacy and the optimal application protocols for enamel deproteinization.

## Data Availability

The data that support the findings of this study are available from the corresponding author upon reasonable request.

## Abbreviations

HOCl: Hypochlorous acid
NaOCl: Sodium hypochlorite
SPSS: Statistical Package for the Social Sciences
ICDAS: International Caries Detection and Assessment System
LED: Light Emitting Diode

## Declarations

This work was accepted as a thesis on May 12, 2025.

## References

[1] Ripa LW. Sealants revisted: an update of the effectiveness of pit-and-fissure sealants. Caries Res. 1993;27(Suppl 1):77–82. 8500131 10.1159/000261608

[2] Salama FS, Al-Hammad NS. Marginal seal of sealant and compomer materials with and without enameloplasty. Int J Paediatr Dent. 2002;12(1):39–46. 11853247

[3] Buonocore MG. A simple method of increasing the adhesion of acrylic filling materials to enamel surfaces. J Dent Res. 1955;34(6):849–53. 13271655 10.1177/00220345550340060801

[4] Futatsuki M, Kubota K, Yeh YC, Park K, Moss SJ. Early loss of pit and fissure sealant: a clinical and SEM study. J Clin Pediatr Dent. 1995;19(2):99–104. 7577741

[5] Burrow MF, Burrow JF, Makinson OF. Pits and fissures: etch resistance in prismless enamel walls. Aust Dent J. 2001;46(4):258–62. 11838872 10.1111/j.1834-7819.2001.tb00289.x

[6] Espinosa R, Valencia R, Uribe M, Ceja I, Saadia M. Enamel deproteinization and its effect on acid etching: an in vitro study. J Clin Pediatr Dent. 2008;33(1):13–9. 19093646 10.17796/jcpd.33.1.ng5462w5746j766p

[7] Valencia R, Espinosa R, Borovoy N, Pérez S, Ceja I, Saadia M. Deproteinization effectiveness on occlusal enamel surfaces and resultant acid etching patterns: an in vitro study. J Clin Pediatr Dent. 2018;42(6):434–41. 30085877 10.17796/1053-4625-42.6.5

[8] Harleen N, Ramakrishna Y, Munshi AK. Enamel deproteinization before acid etching and its effect on the shear bond strength–an in vitro study. J Clin Pediatr Dent. 2011;36(1):19–23. 22900439 10.17796/jcpd.36.1.l345875912750624

[9] Justus R, Cubero T, Ondarza R, Morales F. A new technique with sodium hypochlorite to increase bracket shear bond strength of fluoride-releasing resin-modified glass ionomer cements: comparing shear bond strength of two adhesive systems with enamel surface deproteinization before etching. Semin Orthod. 2010;16:66–75.

[10] Mainnemare A, Mégarbane B, Soueidan A, Daniel A, Chapple IL. Hypochlorous acid and taurine-N-monochloramine in periodontal diseases. J Dent Res. 2004;83(11):823–31. 15505230 10.1177/154405910408301101

[11] Fukuzaki S. Mechanisms of actions of sodium hypochlorite in cleaning and disinfection processes. Biocontrol Sci. 2006;11(4):147–57. 17190269 10.4265/bio.11.147

[12] Boecker D, Zhang Z, Breves R, Herth F, Kramer A, Bulitta C. Antimicrobial efficacy, mode of action and in vivo use of hypochlorous acid (HOCl) for prevention or therapeutic support of infections. GMS Hyg Infect Control. 2023;18:Doc07. 37034111 10.3205/dgkh000433 PMC10073986

[13] Pavan R, Jain S, Shraddha, Kumar A. Properties and therapeutic application of bromelain: a review. Biotechnol Res Int. 2012;2012:976203. 23304525 10.1155/2012/976203 PMC3529416

[14] Liliany D, Widyarman A, Erfan E, Sudiono J, Sadono M. Enzymatic Activity of Bromelain Isolated Pineapple (Ananas comosus) Hump and Its Antibacterial Effect on Enterococcus faecalis. Sci Dent J. 2018;2:41.

[15] Hasija P, Sachdev V, Mathur S, Rath R. Deproteinizing agents as an effective enamel bond enhancer-an in vitro study. J Clin Pediatr Dent. 2017;41(4):280–3. 28650791 10.17796/1053-4628-41.4.280

[16] Paing SY, Tichy A, Hosaka K, Nagano D, Nakajima M, Tagami J. Effect of smear layer deproteinization with HOCl solution on the dentin bonding of conventional and resin-modified glass-ionomer cements. Eur J Oral Sci. 2020;128(3):255–62. 32311176 10.1111/eos.12694

[17] Polat S, Cinar C. The effect of the use of the deproteinization agent hypochlorous acid and two different pit and fissure sealant self-adhesive flowable composites upon its bonding with the enamel. J Clin Pediatr Dent. 2024;48(1):144–51. 38239167 10.22514/jocpd.2024.016

[18] Sisman KY, Baltaci E, Ozveren N. The effect of different deproteinization agents on microleakage and penetration depth of fissure sealants in permanent molars: An in vitro study. Contemp Pediatr Dentistry. 2023;4(3):97–104.

[19] Gale MS, Darvell BW. Thermal cycling procedures for laboratory testing of dental restorations. J Dent. 1999;27(2):89–99. 10071465 10.1016/s0300-5712(98)00037-2

[20] Miyazaki M, Sato M, Onose H, Moore BK. Influence of thermal cycling on dentin bond strength of two-step bonding systems. Am J Dent. 1998;11(3):118–22. 9823072

[21] De Munck J, Van Landuyt K, Coutinho E, Poitevin A, Peumans M, Lambrechts P, Van Meerbeek B. Micro-tensile bond strength of adhesives bonded to Class-I cavity-bottom dentin after thermo-cycling. Dent Mater. 2005;21(11):999–1007. 16181669 10.1016/j.dental.2004.11.005

[22] Zhou Y, Huang X, Wu L, Liang Y, Huang Y, Huang S. Microleakage, microgap, and shear bond strength of an infiltrant for pit and fissure sealing. Heliyon. 2023;9(5):e16248. 37229157 10.1016/j.heliyon.2023.e16248 PMC10205491

[23] Navazesh M, Kumar SK. Measuring salivary flow: challenges and opportunities. J Am Dent Assoc. 2008;139(Suppl):s35–40. 10.14219/jada.archive.2008.0353 18460678

[24] Zakzuk Alshahni R, Sato K, Hosaka K, Hatayama T, Chiba A, Foxton RM, Tagami J, Sumi Y, Shimada Y, Nakajima M. Effect of smear layer deproteinization with enzyme solutions on bonding efficacy of one-step self-etch adhesives. Int J Adhes Adhes. 2020;102.

[25] Gómez S, Bravo P, Morales R, Romero A, Oyarzún A. Resin penetration in artificial enamel carious lesions after using sodium hypochlorite as a deproteinization agent. J Clin Pediatr Dent. 2014;39(1):51–6. 25631727 10.17796/jcpd.39.1.e72570275387527r

[26] Rishika, Garg N, Mayall SS, Pathivada L, Yeluri R. Combined effect of enamel deproteinization and intermediate bonding in the retention of pit and fissure sealants in children: a randomized clinical trial. J Clin Pediatr Dent. 2018;42(6):427–33. 30085876 10.17796/1053-4625-42.6.4

[27] Fernandez-Barrera MA, da Silva AF, Pontigo-Loyola AP, Zamarripa-Calderon JE, Piva E, Cuevas-Suarez CE. The effect of deproteinizing agents on bond strength of resin-based materials to enamel: a systematic review and meta-analysis of in vitro studies. J Adhes Dent. 2021;23(4):287–96. 34269539 10.3290/j.jad.b1649893

[28] Bhoomika ARY. Enamel Deproteinization After Acid Etching - Is It Worth The Effort. Dentistry. 2014;04(02).

[29] Ahuja B, Yeluri R, Baliga S, Munshi AK. Enamel deproteinization before acid etching–a scanning electron microscopic observation. J Clin Pediatr Dent. 2010;35(2):169–72. 21417119 10.17796/jcpd.35.2.9gw7147381836380

[30] Khatib MS, Devarasanahalli SV, Aswathanarayana RM, Venkateswara AH, Nadig RR. Microtensile bond strength of composite resin following the use of bromelain and papain as deproteinizing agents on etched dentin: an in vitro study. Int J Clin Pediatr Dent. 2020;13(1):43–7. 32581478 10.5005/jp-journals-10005-1743 PMC7299883

[31] Chauhan K, Basavanna RS, Shivanna V. Effect of bromelain enzyme for dentin deproteinization on bond strength of adhesive system. J Conserv Dent. 2015;18(5):360–3. 26430297 10.4103/0972-0707.164029 PMC4578178

[32] Pitchika V, Birlbauer S, Chiang ML, Schuldt C, Crispin A, Hickel R, Kühnisch J. Shear bond strength and microleakage of a new self-etch adhesive pit and fissure sealant. Dent Mater J. 2018;37(2):266–71. 29279545 10.4012/dmj.2017-072

